# Suicide

**Published:** 1889-08

**Authors:** S. H. Preston


### SUICIDE.

BY S. H. PRESTON.

"Murder is a very bad habit," wrote the boy who wrestled with the sanguinary subject in his first composition. And humanity seems to be getting into quite a habit of suicide. The custom of "shuffling off" is growing more general in this generation than any by-gone one. Instances of individuals dodging through some side-door of death are increasing at a startling rate.

Sometimes there seems to be an epidemic of suicide. It is stated there have been more cases of *felo-de se* in the last six months than in any previous six years. The daily press teems with dismal details of self-destruction by rope and razor, revolver and "rough on rats." Pulpits resound with rhetorical outbursts of dismay. Statesmen and social scientists are studying the astounding statists, and legislative Solons seek to stop the spread of the naughty practice by special statue. They have even gone so far as to prohibit people by Penal Code from killing themselves. They declare it to be as unlawful to abscond from this life as from a judgment creditor. It is pronounced an offence against the citizens of this world to pass from it precipitately. Still, languishing lovers look to laudanum and belladonna for surcease of sorrow, and despairing sewing women spend their last cent for Paris green. And there are miserable mothers with no more respect for the Penal Code than to gather their little ones about them and turn on the gas.

Now, the greatest ground for wonder is, not that so many, but that so few, seek an outlet from this life of turmoil and trouble. It is surprising that the majority of mankind do not do themselves away. So much bitter disappoint and heart-breaking bereavement, so much unbearable pain and grief and remorse. Life at best is something fearful—its frustrated affections and fleeting felicities, its fading flowers and fatal follies, its falseness, afflictions, and fruits that turn to ashes when tasted. Fretting after last illusions, following *ignis fatui*, fighting the flesh and the unfriendly forces of nature. Why, only patches of our planet are habitable, and even there, life is a stern struggle for subsistence. Everywhere the strong preying upon the weak, the weak upon the still weaker. O, it is a tragedy too terrible for tears, and it is strange that so many delay for Death to ring down the curtain. Only the thrust of a knife, the twitch of a trigger, a jump from the abutment of a bridge, a moment's twinge no more than the toothache, and the link of earthly suffering is snapped forever.

But our compendium of legislative wisdom, called Penal Code, classes suicide a crime, or rather prescribes punishment for not consummating it successfully. It works somewhat like the old time water test for witches. If the suspected swam she was killed as a witch, and if she sank she was drowned. For one who would elude the hardships of life and makes a botch of it, the Penal Code offers the climax of all hardships—the horrors of a prison, generally worse than death. It thus serves as an inducement for a thorough job.

This Penal Code wonld have worked rather roughly with William Cowper, the pious poet and Christian hymnologist, author of "Oh for a closer walk with God," "There is a fountain filled with tears," "What various hindrances we meet," who made three failures to take his life; first, in the Thames, from which he was rescued by a man on the bank; second, that same night by the breaking of his knife upon which he had thrown himself; and third, by the parting of the rope with which he had hanged himself. Well for William that he is not doing such things now in New York.

And there was another celebrated Christian, a man of pre-eminent piety, the greatest scientist and intellectual giant of his generation, one who elicited the utmost admiration of such brother scientists as Buckland and Murchison, theologians like Chalmers, and the universities of Christendom,—Hugh Miller, the grand geologist of Scotland, immortalized by his two books, "Footprints of the Creator," and "Testimony of the Rocks." Hugh Miller blotted out his own brilliant brain with a bullet, and was found dead with a revolver by his side. Were this grand student of the rocks now here and made a miss with his revolver, he would be handcuffed as a criminal and hustled off to Sing Sing to break stone for the state.

According to our Penal Code many of the great and good and admired men of all the ages became criminals when they stepped off the shore of life—Isocrates, who killed himself rather than surrender to Philip of Macedon; Demasthenes, who poisoned himself rather than surrender to Alexander's ambassador; Cato, the incorruptible, who, after inflicting upon himself fatal wounds, tore them open three times and bled to death rather than submit to Cæsar; Mithridates, who perished by his own hand rather than render homage to Pampey; Hannibal, who destroyed himself with poison concealed in his ring; Lycurgus, the Grecian Lawgiver, Brutus, and too many more to mention in this place, who committed suicide. Napoleon tried to terminate his own career after the Moscow disaster by drinking a deadly opiate; and was only saved by the vigilance of a servant who, hearing his master arise in the night and mix something in a glass, suspected its nature, and with the help of hastily summoned medical skill, worked over him till his resuscitation was effected. Only saved for St. Helena and a slow and cruel death from cancer in the stomach.

"It would be no crime for me to divert the Nile or the Danube from its natural bed. Where, then, can be the crime in my diverting a few drops of blood from their ordinary channel?" Thus wrote David Hume, the historian. If a crime, it certainly is one committed and commended by the most honored heroes of history, by patriots and philosophers, the virtuous and high-souled in all ages; the Stoics of ancient Greece, Seneca, Cassius, Arria, Atticus, Pliny, Petronicus, Arbiter, and half the great dramatists and poets, ancient and modern; approved by Pope, Thomas Moore, the Christian Addison, and eminent men too numerous to name. Eustice Budgell, a relative of Addison, who threw himself into the Thames, left on his table Addison's celebrated tragedy of "Cato," open at the noble Roman's soliloquy, on a slip of paper these lines, "What Cato did and Addison approved, must needs be right."

Suicide was once held to be an honorable, a brave and virtuous act by ancient worthies whose memories are still cherished by mankind, and it has been justified and sanctioned by many of the greatest minds of modern times. Strange that the Christian world to-day should be so sweeping and severe in denouncing it as a crime when it is not, to our knowledge, anywhere forbidden by their Bible. If it is we would be pleased to be pointed to the passage. The early Church permitted the voluntary provocation of martyrdom; and the Christian maidens and matrons who, like St. Pelagia, took their own lives at the prospect of violation by pagan persecutors, have been canonized. At one time suicide assumed a fanatical phase among the Aldigensian Christians, who sought death by starvation.

There are crimes of omission as well as commission. And if suicide be a crime, may it not be one of the former as well as the latter? Should not a man who can save his life but deliberately declines to do so be considered a suicide? A man is cast upon a railroad crossing. He can get up and walk away if he wills to do so. He hears a train approaching, and instead of running away remains and is crushed into a lifeless mass. Was not his death as willful and certain as though he stabbed himself to the heart?

Now, right here we have some hard questions for Christians who cant so vehemently about the awful and cowardly crime of self-destruction. Was Christ's death on the cross voluntary or not? Yea or Nay. Could he not have saved his life had he so desired? Did he not choose to die when he could have lived? These questions are propounded with due reverence for his imputed divinity, and merely relate to his death as a human being. Whether he were God or son of God, he died as a man. And what Christian dare deny that his death was voluntary? And what is the other name for voluntary death? But he died for others, says the Christian. Granted. But may not a man die for himself without his memory receiving the stigma of criminal? If it be heroic to die for duty, if it be patriotic to die for country, may it not be justifiable or even commendable under certain circumstances to die for self?

Now we do not wish to be understood as sanctioning suicide. We merely maintain that whether it be wrong or not, depends entirely upon circumstances and the purpose that prompts the dead. We can imagine instances in which, instead of its being a rash and atrocious act, it would be both a moral and meritorious one. In the case of a hopeless sufferer, whose prolonged life here is a hell of which none worse hereafter can be conceived, an hourly accumulated agony to himself and a grief to others, we cannot see the sin against himself or against society to abruptly break the brittle band that binds the soul to a body that were better buried. What is the great good of prolonging hopeless human misery?

So we say it were better for both the sufferer and those he burdens to quit the world when he wills. We can stand on any street corner in New York and see wretched specimens of humanity pass, the sight of whom, were we asked what we would do in their condition, would prompt the ready response, "Commit suicide." O, it is a terrible thought that there are so many to whom death would be the only relief, who cannot hope for any alleviation in this life. And as to the human accountability of terminating such a life, we hold that one man has as good right tc go in quest of a better world as another has to go to Europe for his health.

We have said this much to show that death by one's own hand is not necessarily criminal nor ignominious, and does not invariably indicate insanity or sinfulness. We say this in considerate sympathy for those who have lost friends by suicide, that they may not feel as though they were mourning the death of a criminal, and that any one who may have a relative die voluntarily need not feel they should rely upon the "charitable perjury of juries" to make it appear to the coroner that the act was accidental.

